# Supplementary material for: Effective Cryopreservation of a Bioluminescent Auxotrophic Escherichia coli-Based Amino Acid Array to Enable Long-Term Ready-to-Use Applications
Source: Biosensors (Basel). 2021 Jul 26;11(8):252. doi: 10.3390/bios11080252 (PMC8393857; doi:10.3390/bios11080252)
Supplement: Supplementary file 1 [file biosensors-11-00252-s001.zip › biosensors-1304526-supplementary.pdf]

biosensors

Article

Effective Cryopreservation of a Bioluminescent Auxotrophic *Escherichia coli*-Based Amino Acid Array to Enable Long-Term Ready-to-Use Applications

Hee Tae Ahn [1,†], In Seung Jang [2,†], Thinh Viet Dang [1], Yi Hyang Kim [2], Dong Hoon Lee [1], Hyeun Seok Choi [2], Byung Jo Yu [2,*] and Moon Il Kim [1,*]

[1] Department of BioNano Technology, Gachon University, 1342 Seongnamdae-ro, Sujeong-gu, Seongnam 13120, Korea; venice4@naver.com (H.T.A.); dvietthinh96@gmail.com (T.V.D.); dhlee9219@gmail.com (D.H.L.)

[2] Green and Sustainable Materials R&D Department, Research Institute of Clean Manufacturing System, Korea Institute of Industrial Technology (KITECH), Cheonan 31056, Korea; isjang@kitech.re.kr (I.S.J.); kyscent@kitech.re.kr (Y.H.K.); hchoi@kitech.re.kr (H.S.C.)

* Correspondence: bjyu@kitech.re.kr (B.J.Y.); moonil@gachon.ac.kr (M.I.K.); Tel.: +82-41-589-8456 (B.J.Y.); +82-31-750-8563 (M.I.K.);

† These authors contributed equally to this work.

Citation: Ahn, H.T.; Jang, I.S.; Dang, T.V.; Kim, Y.H.; Lee, D.H.; Choi, H.S.; Yu, B.J.; Kim, M.I. Effective Cryopreservation of a Bioluminescent Auxotrophic *Escherichia coli*-Based Amino Acid Array to Enable Long-Term Ready-to-Use Applications. *Biosensors* **2021**, *11*, 252. https://doi.org/10.3390/bios11080252

Received: 2 July 2021
Accepted: 25 July 2021
Published: 26 July 2021

Publisher's Note: MDPI stays neutral with regard to jurisdictional claims in published maps and institutional affiliations.

Copyright: © 2021 by the authors. Licensee MDPI, Basel, Switzerland. This article is an open access article distributed under the terms and conditions of the Creative Commons Attribution (CC BY) license (http://creativecommons.org/licenses/by/4.0/).

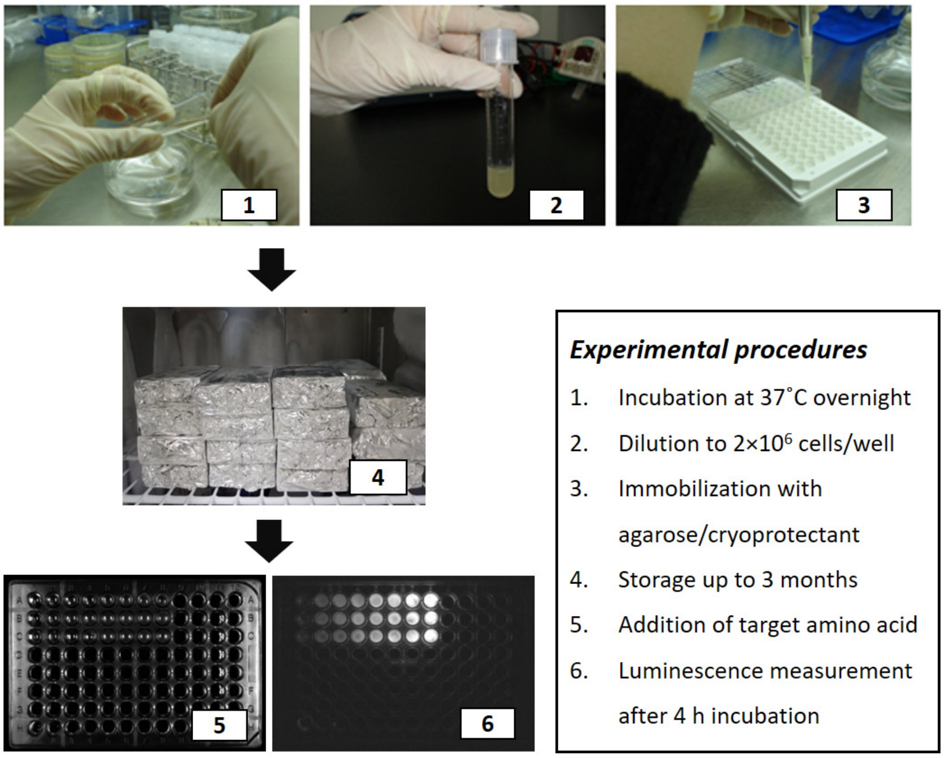

Figure S1. Experimental procedures for ready-to-use amino acid array comprising *E. coli* auxotroph.
